# A restriction-free method for gene reconstitution using two single-primer PCRs in parallel to generate compatible cohesive ends

**DOI:** 10.1186/s12896-017-0346-5

**Published:** 2017-03-17

**Authors:** Fanli Zeng, Zhimin Hao, Pan Li, Yanan Meng, Jingao Dong, Yibin Lin

**Affiliations:** 1 College of Life Sciences, Hebei Agricultural University, Baoding, 071001 China; 2 the University of Texas Houston McGovern Medical School, Houston, 77030 USA

**Keywords:** Restriction-free cloning, Gene reconstitution, Single-primer PCR, High-throughput cloning

## Abstract

**Background:**

Restriction-free (RF) cloning, a PCR-based method for the creation of custom DNA plasmids, allows for the insertion of any sequence into any plasmid vector at any desired position, independent of restriction sites and/or ligation. Here, we describe a simple and fast method for performing gene reconstitution by modified RF cloning.

**Results:**

Double-stranded inserts and acceptors were first amplified by regular PCR. The amplified fragments were then used as the templates in two separate linear amplification reactions containing either forward or reverse primer to generate two single-strand reverse-complement counterparts, which could anneal to each other. The annealed inserts and acceptors with 5’ and 3’ cohesive ends were sealed by ligation reaction. Using this method, we made 46 constructs containing insertions of up to 20 kb. The average cloning efficiency was higher than 85%, as confirmed by colony PCR and sequencing of the inserts.

**Conclusions:**

Our method provides an alternative cloning method capable of inserting any DNA fragment of up to at least 20 kb into a plasmid, with high efficiency. This new method does not require restriction sites or alterations of the plasmid or the gene of interest, or additional treatments. The simplicity of both primer design and the procedure itself makes the method suitable for high-throughput cloning and structural genomics.

**Electronic supplementary material:**

The online version of this article (doi:10.1186/s12896-017-0346-5) contains supplementary material, which is available to authorized users.

## Background

The manipulation of recombinant DNA molecules is an indispensable step in modern high-throughput protein crystallization studies [1]. Restriction enzyme/ligase cloning, which relies on restriction enzyme digestion and ligation, is a simple and easy way to move a fragment of double-stranded DNA from one plasmid to another [2]. However, this technique has two limitations [3]: it is ineffective when lack of unique restriction sites and sometimes results in introduction of unwanted extra sequences. To circumvent these limitations, various restriction endonuclease cleavage site–independent cloning methods have recently been developed [4–21]. These methods have made cloning more accessible in cases in which conventional restriction site cloning was difficult or impossible. While as alternative cloning strategies are still required for more choices.

The restriction-free (RF) cloning strategy, as described extensively in the literature, was developed as a powerful tool for reconstituting genes in circular vectors [3, 22]. Because RF cloning requires no alterations in the plasmid or the gene of interest, it is exceptionally well-suited for high-throughput cloning. The gene of interest is amplified in a regular polymerase chain reaction (PCR), which produces a primer pair that, once annealed to the vector of interest, is extended in a linear amplification reaction. Thus, this method relies on amplified genes functioning as primers. However, this approach also has limitations. First, the motion of large DNA fragments and the formation of secondary structures will affect the efficiency of PCR. Second, this method relies on digestion with *Dpn*I, which cleaves methylated DNA, to remove parental plasmids. The efficiency of *Dpn*I treatment is influenced by many factors, and requires vector propagation in Dam^+^ strains [23].

In this paper, we describe a simple and fast method for performing gene reconstitution by modified restriction-free (MRF) cloning. In this method, two rounds of PCR generate two DNA fragments with compatible 5' or 3’ cohesive ends, which are therefore able to ligate to each other. This new method is independent of the existence of restrictions sites and *Dpn*I treatment. Using this method, we made 46 constructs with inserts of variable size, with average cloning efficiency higher than 85%. The efficiency was not significantly affected by the insert length up to 20 kb.

## Results

### Method overview and primer design

**Fig. 1:**
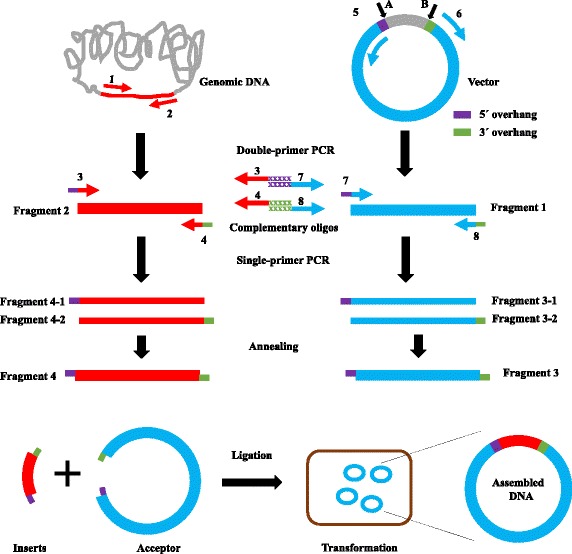
Schematic representation of MRF cloning. The insert gene or vector was amplified by regular double-primer PCR using genomic DNA, cDNA, or the original vector as template. Compatible cohesive ends of insert gene or vector were created by two single-primer linear PCRs performed in parallel, followed by annealing of the two PCR products. Inserts and acceptors with compatible cohesive ends were then assembled by ligation

Figure 1 shows the scheme for MRF cloning. We define the 5–8 bp DNA fragments before the insert site A as “5’ overhang” and those after insert site B as “3’ overhang” (see Fig. 1, Additional file 1: Figure S1 and Additional file 2: Figure S2). To replace a gene (Fig. 1, red line) in a vector between sites A and B, we designed eight primers (Additional file 3: Table S1): primer 1, forward primer, which contains a ~25 bp sequence homologous to the positive strand of the gene; primer 2, reverse primer, which contains a ~25 bp sequence homologous to the negative strand of the gene; primer 3, forward primer, which is the same as primer 1 but has an additional 5’ overhang at the 5’ end; primer 4, reverse primer, which is the same as primer 2 but has an additional 3’ overhang at the 5’ end; primer 5, forward primer, which contains a ~25 bp sequence homologous to the negative strand of the vector; primer 6, reverse primer, which contains a ~25 bp sequence homologous to the positive strand of the vector; primer 7, reverse primer, which is the same as primer 5 but has an additional 5' overhang at the 5’ end; primer 8, reverse primer, which is the same as primer 6 but has an additional 3’ overhang at the 5’ end. All primers were designed with G or C as the 5’ and 3’ terminal nucleotide.

We used two pairs of primers, primer 1/primer 2 and primer 5/primer 6, to generate two DNA fragments, using the target gene and vector as templates, respectively. The resultant PCR products were gel purified. We then amplified these two DNA fragments in two separate PCR reactions containing either forward or reverse primer, which will add 5’ overhang or 3’ overhang to the PCR products (Fig. 1). Finally, DNA fragments with complementary overhang at the 5’ or 3’ end were able to anneal to each other, and were joined by DNA ligase (Fig. 1). The ligated products were then transformed into DH5α competent cells. The inserted genes were verified by colony PCR and further confirmed by DNA sequencing.

### MRF cloning can assemble insert DNA fragment into target vector

We first tested this protocol to reconstitute the *E. coli radA* gene into pET22b. Based on the initial success of the protocol, we continued to employ it to generate the constructs needed for our studies. For example, we planned to replace the *radA* gene in vector pET22b between the start codon ATG (289) and the sequence CACCACCACCACCACCAC (157) (Fig. 1 and Additional file 1: Figure S1) to yield a new construct with the gene under the control of a T7 promoter and a C-terminal His_6_-tag to facilitate protein purification. As shown in Fig. 1, to replace the *radA* gene in the vector, two parallel PCRs were performed to amplify each DNA fragment using the primer pairs pet22b1/pet22b2 and radA1/radA2, as shown in Additional file 3: Table S1, using pET22b or *E. coli* genomic DNA as templates to generate DNA fragments “1” and “2”. Amplified products were separated by 1% agarose gel electrophoresis and purified by gel extraction. We then performed two separate single-primer linear PCRs: (1) using pet22b3 or pet22b4 alone, with DNA fragment “1” as the template, to obtain single-strand DNA fragment “3-1” or “3-2”; and (2) using radA3 or radA4 alone, with DNA fragment “2” as template, to obtain single-strand DNA fragment “4-1” or “4-2”. Fragments “3-1” and “3-2” and “4-1” and “4-2” were then annealed to obtain double-strand DNA fragments “3” and “4”, which have sticky ends that can ligate with other compatible ends. For single-primer PCR, we used ~500 ng of template, about 10 times more than the standard amount recommended for double-primer PCR (Table 1), as DNA amplification in single-primer PCR is linear (only 30-fold for 30 PCR cycles). As the efficiency of cohesive ligation is higher than that of blunt-end ligation, the parental DNA of the products of second-round PCR did not need to be removed. After PCR purification, these second-round PCR products were ready for ligation. In each transformation, we routinely checked eight colonies at random from each transformation by colony PCR with a forward primer annealing to vector and a reverse primer annealing to the inserted gene.

**Table 1:** PCR reaction components

	Reaction 1	Reaction 2a	Reaction 2b
Template DNA	~50 ng	~500 ng	~500 ng
Forward primer (100 μM)	0.25 μL	0.25 μL	
Reverse primer (100 μM)	0.25 μL		0.25 μL
Phusion GC Buffer (5×)	10 μL	10 μL	10 μL
dNTPs (10 mM)	1 μL	1 μL	1 μL
DMSO (100%)	1.5 μL	1.5 μL	1.5 μL
Phusion High Fidelity DNA Polymerase	1 μL	1 μL	1 μL
Add water to	50 μL	50 μL	50 μL

**Fig. 2:**
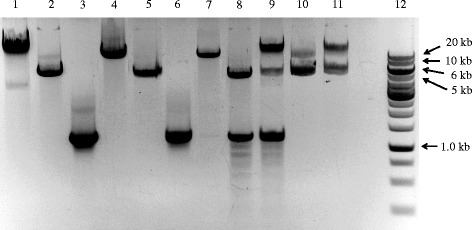
Gel electrophoresis separation of double-primer and single-primer PCR products. 1: Parental plasmid pET22b alone; 2: PCR product from reaction with primers pet22b1/pet22b2 using plasmid pET22b as template; 3: PCR product from reaction with primers radA1/radA2 using *E. coli* genomic DNA as template; 4: PCR product from reaction with primers GeneCluster3-1/GeneCluster3-2 using *E. coli* genomic DNA as template; 5: annealed PCR products from two single-primer linear reactions using primer pet22b3 or pet22b4, and the DNA sample from lane 2 as template; 6: annealed PCR products from two single-primer linear reactions using the primer radA2fw or radA2rv, and the DNA sample from lane 3 as template; 7: annealed PCR products from two single-primer linear reactions using the primer GeneCluster3-3 or GeneCluster3-4, and the DNA sample from lane 4 as template; 8: mixture of DNA samples from lanes 5 and 6 in a molar ratio of 1:3, ready for ligation; 9: mixture of DNA samples from lanes 5 and 6 in a molar ratio of 1:3, after ligation; 10: mixture of DNA samples from lanes 5 and 7 in a molar ratio of 6:1, ready for ligation; 11: mixture of DNA samples from lanes 5 and 7 in a molar ratio of 6:1, after ligation; 12: DNA ladder. PCR products were purified using a QIAquick purification kit (Qiagen) and electrophoresed in 1% agarose with Tris-acetate (40 mM Tris, 20 mM sodium acetate, 1 mM EDTA, pH 8.0) as the running buffer

**Fig. 3:**
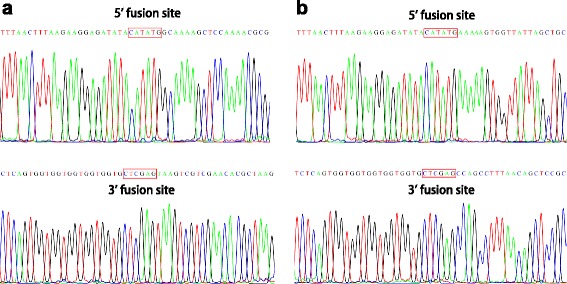
DNA sequencing reveals that genes were correctly placed in the plasmid. **a** Upper panel: DNA sequencing results of the forward cloning site of pET22b-*radA*; lower panel: DNA sequencing results of the reverse cloning site of pET22b-*radA*. **b** Upper panel: DNA sequencing results of the forward cloning site of pET22b-gene cluster 3; lower panel: DNA sequencing results of the reverse cloning site of pET22b-gene cluster 3

The agarose gel in Fig. 2 shows the DNA products of one sample at successive steps of our procedure. Plasmid alone, prior to PCR, shows two major bands (Fig. 2, lane 1). After the double-primer PCR with forward (F) and reverse (R) primers, major bands can be seen at the expected sizes corresponding to the PCR-synthesized linear DNA (Fig. 2, lanes 2 and 3). Single-primer PCR with forward (F) or reverse (R) yielded bands at the expected size of ~5.5 kb (Fig. 2, lane 5) and ~1.4 kb (Fig. 2, lane 6) after annealing the products of single-primer PCR, representing the PCR-synthesized linear DNA with cohesive ends. Additional smaller bands represent non-specific PCR products or single-strand DNA. The ligation of insert into plasmid vector is performed by T4 DNA ligase using a molar ratio of 1:3 vector to insert. As shown in Fig. 2, lane 8 (before ligation) and 9 (after ligation), insert and vector were ligated to one another and shifted to a higher molecular weight. The inserted genes were amplified by colony PCR. The presence of forward and reverse cloning sites were confirmed by DNA sequencing (Fig. 3a).

### Long DNA fragment cloning

To test the suitability of our method for a large DNA fragment, we used it to insert a 20 kb DNA fragment from the *E. coli* genome (200485-220925, Gene Cluster 3, Table 2 and Additional file 4: Table S4) containing 21 genes into pET22b between the start codon ATG (289), and the sequence CACCACCACCACCACCAC (157) (Additional file 1: Figure S1). Gene Cluster 3 was amplified using the primer pair GeneCluster3-1/GeneCluster3-2, as shown in Additional file 4: Table S4, using *E. coli* genomic DNA as template (Fig. 2, lane 4). Gene Cluster 3 with a sticky end was generated by single-primer PCR, as shown in Fig. 1, using primer GeneCluster3-3 or GeneCluster3-4 (Additional file 4: Table S4) (Fig. 2, lane 7). The ligation of Gene Cluster 3 DNA fragment into pET22b was performed with T4 DNA ligase using a molar ratio of vector to insert at 6:1. As shown in Fig. 2, lane 10 (before ligation) and 11 (after ligation), insert and vector were ligated to one another and shifted to a higher molecular weight. The inserted genes were amplified by colony PCR. The presence of forward and reverse cloning sites were confirmed by DNA sequencing (Fig. 3b).

**Table 2:** Genes cloned and efficiency of reconstitution

Gene	Gene ID	Length (bp)	Vector	Positive^a^
*yaiS*	946967	558	pET22b	8 (7)
*ybaY*	945070	574	pET22b	8 (8)
*nfnB*	945778	654	pET22b	8 (6)
*yohK*	949125	696	pET22b	8 (8)
*rlmB*	948694	732	pET22b	8 (8)
*nhoA*	947251	846	pET22b	8 (7)
*dapA*	946952	879	pET22b	8 (8)
*deoC*	948902	780	pET22b	8 (6)
*deoA*	948901	1,230	pET22b	8 (5)
*deoB*	948910	1,224	pET22b	8 (8)
*deoD*	945654	721	pET22b	8 (7)
*yjjJ*	944883	1,332	pET22b	8 (8)
*lplA*	944865	1,017	pET22b	8 (8)
*ytjB*	946089	645	pET22b	8 (7)
*serB*	948913	969	pET22b	8 (8)
*radA*	948912	1,383	pET22b	8 (7)
*yhdP*	2847740	3,801	pET22b	8 (4)
*yjjK*	948909	1,668	pET22b	8 (8)
*slt*	948908	1,938	pET22b	8 (8)
*yjiQ*	948866	561	pET22b	8 (6)
*yjiR*	949089	1,413	pET22b	8 (8)
*yjiS*	948903	165	pET22b	8 (8)
*yjiT*	945056	1,503	pET22b	8 (6)
*yjiV*	2847669	2,937	pET22b	8 (7)
*mcrC*	948880	1,047	pET22b	8 (8)
*mcrB*	949122	1,380	pET22b	8 (6)
*yjiW*	7157066	399	pET22b	8 (8)
*hsdS*	948867	1,395	pET22b	8 (8)
*hsdM*	948872	1,590	pET22b	8 (8)
*hsdR*	948878	3,513	pET22b	8 (5)
*mrr*	948898	915	pET22b	8 (8)
*yjiA*	948882	957	pET22b	8 (8)
*recB*	947286	3,543	pET22b	8 (5)
*yjiY*	948914	2,151	pET22b	8 (8)
*tsr*	948884	1,656	pET22b	8 (8)
*yjiZ*	948879	1,362	pET22b	8 (7)
*yjjM*	7159433	915	pET22b	8 (8)
*yjjN*	7159438	1,023	pET22b	8 (8)
*mdoB*	7159439	2,292	pET22b	8 (4)
*yeeJ*	946498	7,077	pET22b	8 (6)
*gasT*	2520	306	pcDNA™ 3.1	8 (8)
*mcm6*	4175	2,466	pcDNA™ 3.1	8 (6)
*slc18a2*	6571	1,545	pcDNA™ 3.1	8 (7)
Gene cluster 1		10 kb	pET22b	8 (5)
Gene cluster 2		15 kb	pET22b	8 (6)
Gene cluster 3		20 kb	pET22b	8 (4)

^a^Number of colonies checked (number of positive colonies)

### Application of MRF cloning in genes reconstitution

In routine application of our cloning method, we created 46 constructs from *E. coli* genomic DNA and human cDNA (Clontech) with inserts of variable size (Table 2). *E. coli* genes and gene clusters (Additional file 5: Table S2, Additional file 6: Table S3, and Additional file 4: Table S4) were cloned from the *E. coli* genome. The *E. coli* genes and gene clusters were subcloned into pET22b, with the gene under the control of a T7 promoter, and with a C-terminal His_6_-tag to facilitate protein purification (Additional file 1: Figure S1). Human genes were subcloned into the expression vector pcDNA™ 3.1 (+) (Invitrogen) (Additional file 2: Figure S2). Under our test conditions, we achieved an average cloning efficiency of 86.9%. DNA sequencing revealed that all genes were correctly placed in the plasmid.

## Discussion

In this study, we describe a new cloning method. The technique uses two rounds of PCR to obtain inserts and acceptors with compatible cohesive ends, which are then ligated. Using this method, we made 46 constructs with inserts of variable size. The average cloning efficiency was 86.9%, as determined by colony PCR and sequencing of the cloned genes. Because the method relies on PCR to generate cohesive 5’ or 3’ ends for DNA ligation, restriction sites are not needed, which facilitates cloning of the gene of interest. For convenience, we only used the vector pET22b for *E. coli* genes and pcDNA™ 3.1 (+) for human genes, but used inserts of variable size. Our results showed that cloning efficiency was not significantly affected by the different inserts, thus providing a glimpse of the wide choice in inserts that can be used as a template, which then can be used as an alternative method for multiple fragment assembly and library construction.

We noticed that cloning efficiency was not altered dramatically by fragment length. As shown in Table 2 and Additional file 7: Figure S3, this method was suitable for the cloning of large DNA sequences up to 20 kb in size. In contrast to traditional restriction enzyme cloning, the method described here provides a much more flexible approach to gene cloning. Therefore, it represents a cost-effective and simple solution for high-throughput cloning applications. Because this method relies on PCR amplification of the DNA sequences, the most crucial requirement is high-fidelity DNA polymerase. Fortunately, the high-fidelity polymerases recently developed for cloning, e.g., Phusion^®^ High-Fidelity DNA Polymerase and KOD Hot Start DNA Polymerase, have extremely low error rates. Therefore, it is no longer challenging to amplify large DNA fragments for use in our method.

## Conclusions

We developed a novel cloning method that provides an alternative approach to DNA assembly. This method is independent of restriction sites and *Dpn*I treatment, and does not introduce undesired operational sequences at the junctions of functional modules. This new method simplifies complex cloning procedures in which long stretches of DNA can be inserted into circular plasmids in an unrestricted way, and the efficiency does not decrease for long inserts up to 20 kb. The simplicity of both primer design and the procedure itself makes the method suitable for high-throughput studies. The protein of interest is expressed without the addition of extra residues originating from the cloning procedure, making it an attractive alternative method for structural genomics.

## Methods

### Materials

Phusion® High-Fidelity DNA Polymerase, DNA marker, Taq DNA polymerase, and T4 DNA ligase were purchased from New England Biolabs, and cloning kits from Qiagen. pET22b, pcDNA™ 3.1 (+), and host strain *Escherichia coli* DH5α were obtained from Invitrogen. Human cDNAs were purchased from Clontech. Oligonucleotide primers were purchased from Invitrogen. PCR purification and gel extraction kits were purchased from Qiagen. Plasmids were isolated using a QIAprep Spin Miniprep Kit (Qiagen). All other chemicals used in the study were of molecular biology grade.

### Touchdown PCR

PCR reactions were performed to generate DNA fragments in a final volume of 50 μL using Phusion® High-Fidelity DNA Polymerase (New England Biolabs) and the primer pair as shown in Additional file 3: Table S1. After the initial denaturation step at 98 °C for 5 min, the PCR was conducted for 20 cycles with denaturation at 98 °C for 20 s; primer annealing from 60 °C to 50 °C with a step of -0.5 °C each cycle for 20 s; extension at 72 °C for 30 s/kb; and 10 cycles with an annealing temperature at 52 °C. When all cycles were completed, the samples were maintained at 72 °C for 10 min to finish all DNA synthesis.

### Ligation

DNA ligation reactions were performed to fuse DNA fragments in a final volume of 20 μL using T4 DNA ligase (New England Biolabs) following the standard protocol from New England Biolabs. In brief, the longer and shorter DNA fragments were mixed at a molar ratio of 1:3–1:10. The reaction was incubated at room temperature for 2 h. After heat inactivation at 65 °C for 10 min, the reaction was chilled on ice. A 10 μL aliquot of the reaction was used to transform 50 μL of competent cells.

### Colony PCR

For each transformation, eight colonies were selected randomly for colony PCR to verify insertion. The colony PCR included 5 units of Taq DNA polymerase (New England Biolabs) and 1× ThermoPol® Buffer (New England Biolabs) in the presence of 200 μM dNTP, 1 mmol each of a primer from the vector and a primer from the insert gene, and a small amount of cells picked from the colony, all in a final volume of 20 μL. The colony PCR conditions were as follows: 95 °C for 2 min; 25 cycles of 95 °C for 30 s, 50 °C for 30 s, and 68 °C for 1 min/kb; and a final extension at 68 °C for 10 min. Insert-positive constructs were confirmed by DNA sequencing.

### Isolation and purification of total genomic DNA from *E. coli*

Mid-log phase *E. coli* DH5α cells were collected by centrifugation at 4 °C for 10 min. The pellet was resuspended in 190 μL of TE supplemented with 10 μL of 10% SDS and 1 μL of 20 mg/mL protease K, and then incubated at 37 °C for 1 h. After 1 h, 30 μL of 5 M NaCl and 30 μL of CTAB/NaCl were added to the solution, and the sample was incubated at 65 °C for 20 min. After incubation, 300 μL of phenol/chloroform/isoamyl alcohol (25:24:1, v/v) was added, and the sample was immediately mixed and centrifuged at 5000 rpm in a table-top microcentrifuge for 10 min. To the upper (aqueous) phase 300 μL of chloroform/isoamyl alcohol (24:1) was added, which was mixed and centrifuged at 5000 rpm. To the resultant aqueous phase 300 μL of isoamyl alcohol was added; after mixing, the sample was incubated at room temperature for 10 min to precipitate DNA. To pellet DNA, the sample was centrifuged at 5000 rpm for 10 min. The pellet was resuspended in 500 μL of 70% ethanol and centrifuged at 5000 rpm for 10 min. The supernatant was discarded, and the pellet was dried and dissolved in 20 μL of TE buffer.

### Plasmid transformation and isolation

The competent DH5α cells were prepared by calcium chloride method [24]. The ligation product (10 μL) was added directly to 50 μL of competent DH5α cells, incubated for 15 min on ice, heat-shocked at 42 °C for 1 min, and then transferred on ice for 5 min. After adding 500 μL of LB, the cells were incubated in a shaker at 37 °C for 60 min. After incubation, cells were pelleted and resuspended in 100 μL of LB, which was then spread on LB plates containing ampicillin (100 μg/mL). After overnight incubation at 37 °C, eight colonies from each transformation were randomly picked and checked by colony PCR. Plasmids were isolated using the Spin Miniprep kit (Qiagen).

## Additional files

Additional file 1: Figure S1. Map of pET22b cloning region. The blue line shows the 5’ overhang sequence, and the green line shows the 3' overhang sequence. The red arrows, A and B, show the reconstituted sites. (PDF 216 kb)
Additional file 2: Figure S2. Map of pcDNA3.1 (+) cloning region. The blue line shows the 5’ overhang sequence, and the green line shows the 3’ overhang sequence. The red arrows, A and B, show the reconstituted sites. (PDF 202 kb)
Additional file 3: Table S1. Primers used in this study. (DOCX 33 kb)
Additional file 4: Table S4. 20 kb DNA fragments from *E.coli* genome. (DOCX 17 kb)
Additional file 5: Table S2. 10 kb DNA fragments from *E.coli* genome. (DOCX 13 kb)
Additional file 6: Table S3. 15 kb DNA fragments from *E.coli* genome. (DOCX 13 kb)
Additional file 7: Figure S3. Cloning efficiency of selected genes. Data obtained from Table 2. (PDF 67 kb)

## Abbreviations

bp: Base pair
CTAB: Hexadecyltrimethylammonium bromide
dNTP: Mix of 2’-deoxynucleoside 5’-triphosphates
kb: Kilobase
LB: Lysogeny broth.
MRF: Modified restriction-free cloning
PCR: Polymerase chain reaction
RF: Restriction-free cloning
