# Supplementary figures and images for: A restriction-free method for gene reconstitution using two single-primer PCRs in parallel to generate compatible cohesive ends
Source: BMC Biotechnol. 2017 Mar 17;17:32. doi: 10.1186/s12896-017-0346-5 (PMC5356277; doi:10.1186/s12896-017-0346-5)

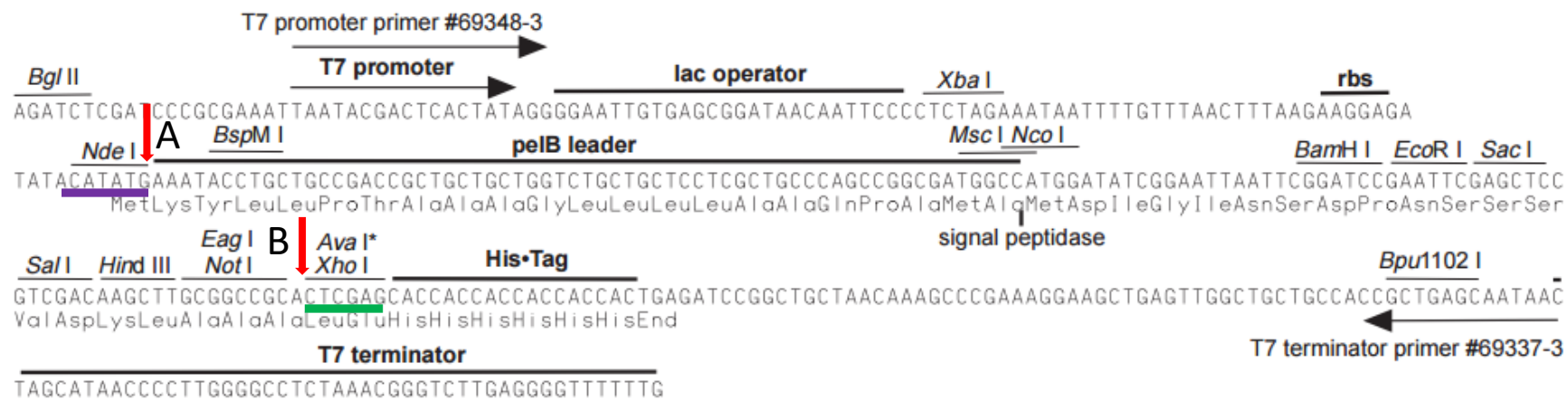

pET-22b(+) cloning/expression region

Supplement: Additional file 1: Figure S1. — Map of pET22b cloning region. The blue line shows the 5’ overhang sequence, and the green line shows the 3' overhang sequence. The red arrows, A and B, show the reconstituted sites. (PDF 216 kb) [file 12896_2017_346_MOESM1_ESM.pdf]

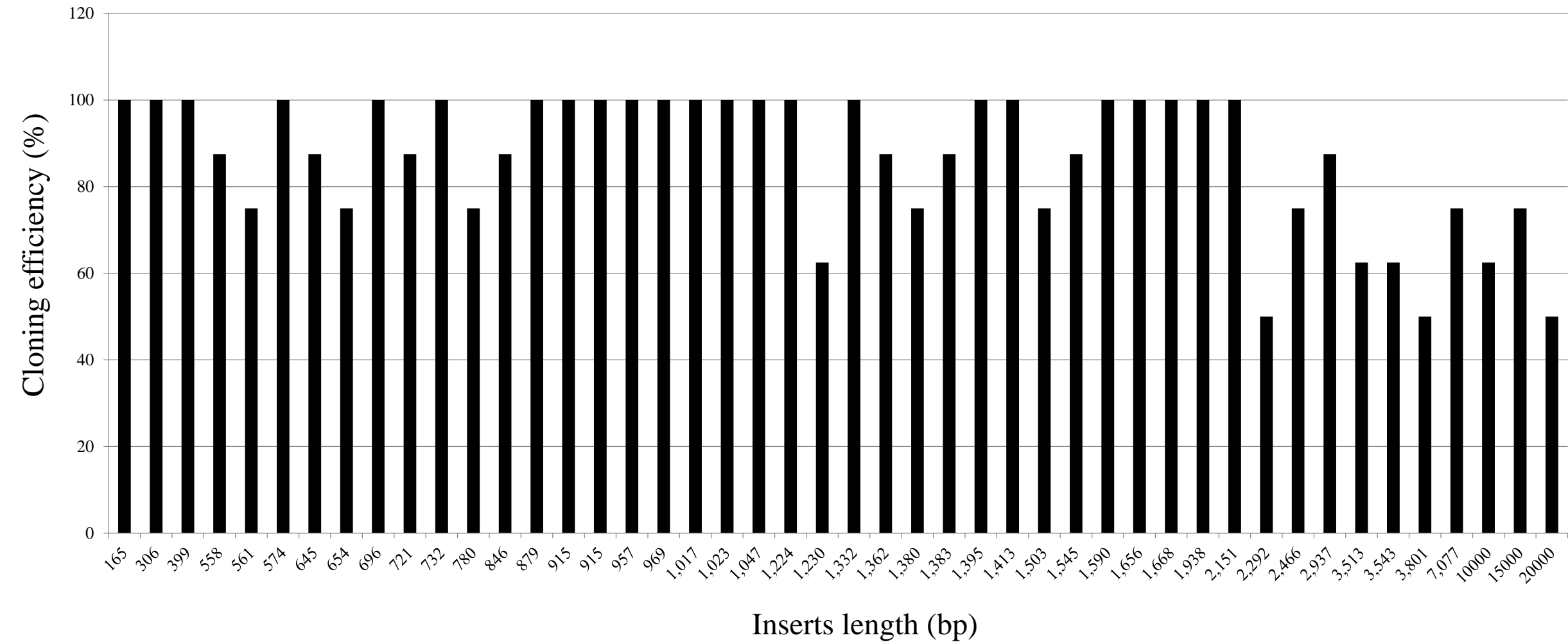

Supplement: Additional file 7: Figure S3. — Cloning efficiency of selected genes. Data obtained from Table 2. (PDF 67 kb) [file 12896_2017_346_MOESM7_ESM.pdf]
