# Supplementary material for: A restriction-free method for gene reconstitution using two single-primer PCRs in parallel to generate compatible cohesive ends
Source: BMC Biotechnol. 2017 Mar 17;17:32. doi: 10.1186/s12896-017-0346-5 (PMC5356277; doi:10.1186/s12896-017-0346-5)
Supplement: Additional file 2: Figure S2. — Map of pcDNA3.1 (+) cloning region. The blue line shows the 5’ overhang sequence, and the green line shows the 3’ overhang sequence. The red arrows, A and B, show the reconstituted sites. (PDF 202 kb) [file 12896_2017_346_MOESM2_ESM.pdf]

```
                 enhancer region (3´ end)
689   CATTGACGTC AATGGGAGTT TGTTTTGGCA CCAAAATCAA CGGGACTTTC CAAAATGTCG

                                CAAT                                 TATA
749   TAACAACTCC GCCCCATTGA CGCAAATGGG CGGTAGGCGT GTACGGTGGG AGGTCTATAT

              3' end of hCMV
                    ↓              putative transcriptional start
809   AAGCAGAGCT CTCTGGCTAA CTAGAGAACC CACTGCTTAC TGGCTTATCG AAATTAATAC

  T7 promoter/primer binding site  Nhe I    A  Pme I  Afl II  Hind III  Asp718 I  Kpn I
869   GACTCACTAT AGGGAGACCC AAGCTGGCTA GCGTTTAAAC TTAAGCTTGG TACCGAGCTC

    BamH I                BstX I*  EcoR I     EcoR V      BstX I*   Not I   Xho I
929   GGATCCACTA GTCCAGTGTG GTGGAATTCT GCAGATATCC AGCACAGTGG CGGCCGCTCG
                               B
     Xba I         Apa I  Pme I           pcDNA3.1/BGH reverse priming site
989   AGTCTAGAGG GCCCGTTTAA ACCCGCTGAT CAGCCTCGAC TGTGCCTTCT AGTTGCCAGC

1049  CATCTGTTGT TTGCCCCTCC CCCGTGCCTT CCTTGACCCT GGAAGGTGCC ACTCCCACTG

              BGH poly (A) site
1109  TCCTTTCCTA ATAAAATGAG GAAATTGCAT
```
