# Supplementary material for: A restriction-free method for gene reconstitution using two single-primer PCRs in parallel to generate compatible cohesive ends
Source: BMC Biotechnol. 2017 Mar 17;17:32. doi: 10.1186/s12896-017-0346-5 (PMC5356277; doi:10.1186/s12896-017-0346-5)
Supplement: Additional file 3: Table S1. — Primers used in this study. (DOCX 33 kb) [file 12896_2017_346_MOESM3_ESM.docx]

**Table S1. Primers used in this study**

| No. | Name | Primer (5'→3')* |
| --- | --- | --- |
| 1 | radA1 | GCAAAAGCTCCAAAACGCGCCTTTG |
| 2 | radA2 | TAAGTCGTCGAACACGCTAAGC |
| 3 | radA3 | **CATATG**GCAAAAGCTCCAAAACGCGCCTTTG |
| 4 | radA4 | **CTCGAG**TAAGTCGTCGAACACGCTAAGC |
| 5 | pet22b1 | TATATCTCCTTCTTAAAGTTAAAC |
| 6 | pet22b2 | CACCACCACCACCACCACTGAGATC |
| 7 | pet22b3 | **CATATG**TATATCTCCTTCTTAAAGTTAAAC |
| 8 | pet22b4 | **CTCGAG**CACCACCACCACCACCACTGAGATC |
| 9 | pcDNA1 | CAGCTTGGGTCTCCCTATAGTGAGTC |
| 10 | pcDNA2 | TGCAGATATCCAGCACAGTGG |
| 11 | pcDNA3 | **GCTAGC**CAGCTTGGGTCTCCCTATAGTGAGTC |
| 12 | pcDNA4 | **GAATTC**TGCAGATATCCAGCACAGTGG |
| 13 | GeneCluster3-1 | AAAAAGTGGTTATTAGCTGCAGGTCT |
| 14 | GeneCluster3-2 | CCAGCCTTTAACAGCTCCGCCGTTAAAC |
| 15 | GeneCluster3-3 | **CATATG**AAAAAGTGGTTATTAGCTGCAGGTCT |
| 16 | GeneCluster2-4 | **CTCGAG**CCAGCCTTTAACAGCTCCGCCGTTAAAC |
| 17 | GeneCluster1-1 | AAATCCGTTTTTACGATTTCCGCCAG |
| 18 | GeneCluster1-2 | AGCCGGTTTTGTTAGCCCAAAAAATTC |
| 19 | GeneCluster1-3 | **CATATG**AAATCCGTTTTTACGATTTCCGCCAG |
| 20 | GeneCluster1-4 | **CTCGAG**AGCCGGTTTTGTTAGCCCAAAAAATTC |
| 21 | GeneCluster2-1 | AAGAACTGGAAAACGCTGCTTCTCG |
| 22 | GeneCluster2-2 | TGACAACTTGACGGCTACATCATTC |
| 23 | GeneCluster2-3 | **CATATG**AAGAACTGGAAAACGCTGCTTCTCG |
| 24 | GeneCluster2-4 | **CTCGAG**TGACAACTTGACGGCTACATCATTC |
| 25 | ybaY1 | AAACTCGTGCACATGGCCAGTGGTTTAG |
| 26 | ybaY2 | ATATTGCGTAGGAGCTGGAACTG |
| 27 | ybaY3 | **CATATG**AAACTCGTGCACATGGCCAGTGGTTTAG |
| 28 | ybaY4 | **CTCGAG**ATATTGCGTAGGAGCTGGAACTG |
| 29 | nfnB1 | GATATCATTTCTGTCGCCTTAAAGC |
| 30 | nfnB2 | CACTTCGGTTAAGGTGATGTTTTG |
| 31 | nfnB3 | **CATATG**GATATCATTTCTGTCGCCTTAAAGC |
| 32 | nfnB4 | **CTCGAG**CACTTCGGTTAAGGTGATGTTTTG |
| 33 | yohK1 | ATGGCGAATATCTGGTGGTCATTAC |
| 34 | yohK2 | GCCCATTACTGCCAGAATAATC |
| 35 | yohK3 | **CATATG**ATGGCGAATATCTGGTGGTCATTAC |
| 36 | yohK4 | **CTCGAG**GCCCATTACTGCCAGAATAATC |
| 37 | rlmB1 | AGCGAAATGATTTACGGCATCCACG |
| 38 | rlmB2 | GCTGCGCTGGCGCACCGCTTC |
| 39 | rlmB3 | **CATATG**AGCGAAATGATTTACGGCATCCACG |
| 40 | rlmB4 | **CTCGAG**GCTGCGCTGGCGCACCGCTTC |
| 41 | nhoA1 | ACGCCCATTCTGAATCACTATTTTG |
| 42 | nhoA2 | TTTTCCCGCCTCCGGGTGCGTATC |
| 43 | nhoA3 | **CATATG**ACGCCCATTCTGAATCACTATTTTG |
| 44 | nhoA4 | **CTCGAG**TTTTCCCGCCTCCGGGTGCGTATC |
| 45 | dapA1 | TTCACGGGAAGTATTGTCGCGATTG |
| 46 | dapA2 | CAGCAAACCGGCATGCTTAAGC |
| 47 | dapA3 | **CATATG**TTCACGGGAAGTATTGTCGCGATTG |
| 48 | dapA4 | **CTCGAG**CAGCAAACCGGCATGCTTAAGC |
| 49 | deoC1 | ACTGATCTGAAAGCAAGCAGCCTGC |
| 50 | deoC2 | GTAGCTGCTGGCGCTCTTACCG |
| 51 | deoC3 | **CATATG**ACTGATCTGAAAGCAAGCAGCCTGC |
| 52 | deoC4 | **CTCGAG**GTAGCTGCTGGCGCTCTTACCG |
| 53 | deoA1 | TTTCTCGCACAAGAAATTATTCG |
| 54 | deoA2 | TTCGCTGATACGGCGATAGACAG |
| 55 | deoA3 | **CATATG**TTTCTCGCACAAGAAATTATTCG |
| 56 | deoA4 | **CTCGAG**TTCGCTGATACGGCGATAGACAG |
| 57 | deoB1 | AAACGTGCATTTATTATGGTGCTGG |
| 58 | deoB2 | GAACATGGCTTTGCCATATTCC |
| 59 | deoB3 | **CATATG**AAACGTGCATTTATTATGGTGCTGG |
| 60 | deoB4 | **CTCGAG**GAACATGGCTTTGCCATATTCC |
| 61 | deoD1 | GCTACCCCACACATTAATGCAGAAATG |
| 62 | deoD2 | CTCTTTATCGCCCAGCAGAACG |
| 63 | deoD3 | **CATATG**GCTACCCCACACATTAATGCAGAAATG |
| 64 | deoD4 | **CTCGAG**CTCTTTATCGCCCAGCAGAACG |
| 65 | yjjJ1 | AGCGAGCTGACTGATCTTTTACTGC |
| 66 | yjjJ2 | CCCGCCCATGCGGGCAACTTTC |
| 67 | yjjJ3 | **CATATG**AGCGAGCTGACTGATCTTTTACTGC |
| 68 | yjjJ4 | **CTCGAG**CCCGCCCATGCGGGCAACTTTC |
| 69 | lplA1 | TCCACATTACGCCTGCTCATCTCTG |
| 70 | lplA2 | CCTTACAGCCCCCGCCATCCATG |
| 71 | lplA3 | **CATATG**TCCACATTACGCCTGCTCATCTCTG |
| 72 | lplA4 | **CTCGAG**CCTTACAGCCCCCGCCATCCATG |
| 73 | ytjB1 | GCTCGCACAAAACTGAAATTCCGGC |
| 74 | ytjB2 | CTCTTTTTTCTCGCTTTCTTCC |
| 75 | ytjB3 | **CATATG**GCTCGCACAAAACTGAAATTCCGGC |
| 76 | ytjB4 | **CTCGAG**CTCTTTTTTCTCGCTTTCTTCC |
| 77 | serB1 | CCTAACATTACCTGGTGCGACCTGC |
| 78 | serB2 | CTTCTGATTCAGGCTGCCTGAG |
| 79 | serB3 | **CATATG**CCTAACATTACCTGGTGCGACCTGC |
| 80 | serB4 | **CTCGAG**CTTCTGATTCAGGCTGCCTGAG |
| 81 | yaiS1 | GATAAGGTTTTAGATTCAGCCCTCC |
| 82 | yaiS2 | GCTTTTATGTTTTTTTAATGCAG |
| 83 | yaiS3 | **CATATG**GATAAGGTTTTAGATTCAGCCCTCC |
| 84 | yaiS4 | **CTCGAG**GCTTTTATGTTTTTTTAATGCAG |
| 85 | yhdP1 | AGGCGATTGCCGGGGATTTTACTGC |
| 86 | yhdP2 | TTGCGCTTTTTCTTTACGCGGTTG |
| 87 | yhdP3 | **CATATG**AGGCGATTGCCGGGGATTTTACTGC |
| 88 | yhdP4 | **CTCGAG**TTGCGCTTTTTCTTTACGCGGTTG |
| 89 | yjjK1 | GCTCAATTCGTTTATACCATGCATCG |
| 90 | yjjK2 | CTTCGCAATACGCTTGTACTTG |
| 91 | yjjK3 | **CATATG**GCTCAATTCGTTTATACCATGCATCG |
| 92 | yjjK4 | **CTCGAG**CTTCGCAATACGCTTGTACTTG |
| 93 | slt1 | GAAAAAGCCAAACAAGTTACCTGGC |
| 94 | slt2 | GTAACGACGTCCCCATTCCGTG |
| 95 | slt3 | **CATATG**GAAAAAGCCAAACAAGTTACCTGGC |
| 96 | slt4 | **CTCGAG**GTAACGACGTCCCCATTCCGTG |
| 97 | yjiQ1 | ACAAACTTCACGACCAGCACGCCGC |
| 98 | yjiQ2 | GCTCTTGAGCCATGAATAGCGC |
| 99 | yjiQ3 | **CATATG**ACAAACTTCACGACCAGCACGCCGC |
| 100 | yjiQ4 | **CTCGAG**GCTCTTGAGCCATGAATAGCGC |
| 101 | yjiR1 | ACGCGTTATCAACATCTGGCGACTC |
| 102 | yjiR2 | TTCCATTGCCCGATACACGGCC |
| 103 | yjiR3 | **CATATG**ACGCGTTATCAACATCTGGCGACTC |
| 104 | yjiR4 | **CTCGAG**TTCCATTGCCCGATACACGGCC |
| 105 | yjiS1 | GAATTTCACGAAAACAGAGCTAAAG |
| 106 | yjiS2 | CTCCACATCCTCCCTGCGTAAC |
| 107 | yjiS3 | **CATATG**GAATTTCACGAAAACAGAGCTAAAG |
| 108 | yjiS4 | **CTCGAG**CTCCACATCCTCCCTGCGTAAC |
| 109 | yjiT1 | GGTCAATCAGAATACATTTCATGGG |
| 110 | yjiT2 | TACATCAATGCTTTGAATATCC |
| 111 | yjiT3 | **CATATG**GGTCAATCAGAATACATTTCATGGG |
| 112 | yjiT4 | **CTCGAG**TACATCAATGCTTTGAATATCC |
| 113 | yjiV1 | CACGTGCTGACAAAACAGCATTACAG |
| 114 | yjiV2 | GGAGGCGATCACCTCCACCAGC |
| 115 | yjiV3 | **CATATG**CACGTGCTGACAAAACAGCATTACAG |
| 116 | yjiV4 | **CTCGAG**GGAGGCGATCACCTCCACCAGC |
| 117 | mcrC1 | GAACAGCCCGTGATACCTGTCCG |
| 118 | mcrC2 | TTTGAGATATTCATCGAAAATG |
| 119 | mcrC3 | **CATATG**GAACAGCCCGTGATACCTGTCCG |
| 120 | mcrC4 | **CTCGAG**TTTGAGATATTCATCGAAAATG |
| 121 | hsdM1 | AACAATAACGATCTGGTCGCGAAGC |
| 122 | hsdM2 | TTCCTTCACCCCACCAAACGC |
| 123 | hsdM3 | **CATATG**AACAATAACGATCTGGTCGCGAAGC |
| 124 | hsdM4 | **CTCGAG**TTCCTTCACCCCACCAAACGC |
| 125 | hsdR1 | ATGAATAAATCCAATTTTGAATTC |
| 126 | hsdR2 | GGCCAGCTCGTCCCAGATATAATC |
| 127 | hsdR3 | **CATATG**ATGAATAAATCCAATTTTGAATTC |
| 128 | hsdR4 | **CTCGAG**GGCCAGCTCGTCCCAGATATAATC |
| 129 | mrr1 | ACGGTTCCTACCTATGACAAATTTATTG |
| 130 | mrr2 | CTCAAAATAGTCCATATCCAGTTTC |
| 131 | mrr3 | **CATATG**ACGGTTCCTACCTATGACAAATTTATTG |
| 132 | mrr4 | **CTCGAG**CTCAAAATAGTCCATATCCAGTTTC |
| 133 | yjiA1 | AACCCGATTGCAGTTACCCTACTCAC |
| 134 | yjiA2 | CTTCCTCAACCCCGCAAACGCAG |
| 135 | yjiA3 | **CATATG**AACCCGATTGCAGTTACCCTACTCAC |
| 136 | yjiA4 | **CTCGAG**CTTCCTCAACCCCGCAAACGCAG |
| 137 | recB1 | AGTGATGTCGCCGAGACACTAGATC |
| 138 | recB2 | CGCCTCCTCCAGGGTCATACCG |
| 139 | recB3 | **CATATG**AGTGATGTCGCCGAGACACTAGATC |
| 140 | recB4 | **CTCGAG**CGCCTCCTCCAGGGTCATACCG |
| 141 | yjiY1 | GATACTAAAAAGATATTCAAGCACATAC |
| 142 | yjiY2 | GTGGTGCGAAGAGATCTTCACG |
| 143 | yjiY3 | **CATATG**GATACTAAAAAGATATTCAAGCACATAC |
| 144 | yjiY4 | **CTCGAG**GTGGTGCGAAGAGATCTTCACG |
| 145 | tsr1 | TTAAAACGTATCAAAATTGTGACCAG |
| 146 | tsr2 | AAATGTTTCCCAGTTCTCCTCG |
| 147 | tsr3 | **CATATG**TTAAAACGTATCAAAATTGTGACCAG |
| 148 | tsr4 | **CTCGAG**AAATGTTTCCCAGTTCTCCTCG |
| 149 | yjiZ1 | GAAAAAGAAAATATCACCATCGATC |
| 150 | yjiZ2 | ATCTTTACGTGGGTCGTTGATC |
| 151 | yjiZ3 | **CATATG**GAAAAAGAAAATATCACCATCGATC |
| 152 | yjiZ4 | **CTCGAG**ATCTTTACGTGGGTCGTTGATC |
| 153 | yjjM1 | AGTCGTTCACAAAATTTACGCCAC |
| 154 | yjjM2 | ATGAGCATAACGCGTGTTCTC |
| 155 | yjjM3 | **CATATG**AGTCGTTCACAAAATTTACGCCAC |
| 156 | yjjM4 | **CTCGAG**ATGAGCATAACGCGTGTTCTC |
| 157 | yjjN1 | TCTACGATGAATGTTTTAATTTGCC |
| 158 | yjjN2 | GAAAGTAATTACACCCTTAATC |
| 159 | yjjN3 | **CATATG**TCTACGATGAATGTTTTAATTTGCC |
| 160 | yjjN4 | **CTCGAG**GAAAGTAATTACACCCTTAATC |
| 161 | mdoB1 | TCAGAACTACTCTCTTTCGCCCTTTTTC |
| 162 | mdoB2 | CCCTTCACGTTCCACAACTTTAATTTC |
| 163 | mdoB3 | **CATATG**TCAGAACTACTCTCTTTCGCCCTTTTTC |
| 164 | mdoB4 | **CTCGAG**CCCTTCACGTTCCACAACTTTAATTTC |
| 165 | yeeJ1 | GCTACGAAGAAGAGAAGTGGAGAAG |
| 166 | yeeJ2 | CAGGTTTTTATAACATGTCGCATAAG |
| 167 | yeeJ3 | **CATATG**GCTACGAAGAAGAGAAGTGGAGAAG |
| 168 | yeeJ4 | **CTCGAG**CAGGTTTTTATAACATGTCGCATAAG |
| 169 | gasT1 | CAGCGACTATGTGTGTATGTGCTGATC |
| 170 | gasT2 | GTTCTCATCCTCAGCACTGCGGCGG |
| 171 | gasT3 | **GCTAGC**CAGCGACTATGTGTGTATGTGCTGATC |
| 172 | gasT4 | **GAATTC**GTTCTCATCCTCAGCACTGCGGCGG |
| 173 | mcm61 | GACCTCGCGGCGGCAGCGGAGCCGG |
| 174 | mcm62 | ATCTTCGAGCAAGTAGTTAGGGTTAAC |
| 175 | mcm63 | **GCTAGC**GACCTCGCGGCGGCAGCGGAGCCGG |
| 176 | mcm64 | **GAATTC**ATCTTCGAGCAAGTAGTTAGGGTTAAC |
| 177 | slc18a21 | GCCCTGAGCGAGCTGGCGCTGGTCC |
| 178 | slc18a22 | GTCACTTTCAGATTCTTCATCTTCACC |
| 179 | slc18a23 | **GCTAGC**GCCCTGAGCGAGCTGGCGCTGGTCC |
| 180 | slc18a24 | **GAATTC**GTCACTTTCAGATTCTTCATCTTCACC |

* bold purple letters show 5' overhangs, and bold green letters show 3' overhangs.
