# Supplementary material for: A restriction-free method for gene reconstitution using two single-primer PCRs in parallel to generate compatible cohesive ends
Source: BMC Biotechnol. 2017 Mar 17;17:32. doi: 10.1186/s12896-017-0346-5 (PMC5356277; doi:10.1186/s12896-017-0346-5)
Supplement: Additional file 4: Table S4. — 20 kb DNA fragments from E.coli genome. (DOCX 17 kb) [file 12896_2017_346_MOESM4_ESM.docx]

**Table S4. 20 kb DNA fragments from *E.coli* genome**

| Gene | JW ID | Direction | Left nt | Right nt |
| --- | --- | --- | --- | --- |
| *hlpA* | JW0173 | + | 200485 | 200946 |
| *lpxD* | JW0174 | + | 200974 | 201975 |
| *fabZ* | JW0175 | + | 202104 | 202535 |
| *lpxA* | JW0176 | + | 202563 | 203327 |
| *lpxB* | JW0177 | + | 203351 | 204475 |
| *rnhB* | JW0178 | + | 204496 | 205068 |
| *dnaE* | JW0179 | + | 205129 | 208587 |
| *accA* | JW0180 | + | 208624 | 209559 |
| *ldcC* | JW0181 | + | 209682 | 211799 |
| *yaeR* | JW0182 | + | 211880 | 212245 |
| *tilS* | JW0183 | + | 212334 | 213608 |
| *rof* | JW0184 | - | 213699 | 213929 |
| *yaeP* | JW0185 | - | 213946 | 214122 |
| *yaeQ* | JW0186 | + | 214294 | 214815 |
| *yaeJ* | JW0187 | + | 214836 | 215234 |
| *nlpE* | JW0188 | + | 215272 | 215958 |
| *yaeF* | JW5016 | - | 216200 | 217000 |
| *proS* | JW0190 | - | 217078 | 218772 |
| *yaeB* | JW0191 | - | 218908 | 219591 |
| *rcsF* | JW0192 | - | 219612 | 219992 |
| *metQ* | JW0193 | - | 220134 | 220925 |
