# Supplementary material for: A restriction-free method for gene reconstitution using two single-primer PCRs in parallel to generate compatible cohesive ends
Source: BMC Biotechnol. 2017 Mar 17;17:32. doi: 10.1186/s12896-017-0346-5 (PMC5356277; doi:10.1186/s12896-017-0346-5)
Supplement: Additional file 5: Table S2. — 10 kb DNA fragments from E.coli genome. (DOCX 13 kb) [file 12896_2017_346_MOESM5_ESM.docx]

**Table S2. 10 kb DNA fragments from *E.coli* genome**

| Gene | JW ID | Direction | Left nt | Right nt |
| --- | --- | --- | --- | --- |
| *yaaI* | JW0012 | - | 11403 | 11783 |
| *dnaK* | JW0013 | + | 12166 | 14058 |
| *dnaJ* | JW0014 | + | 14171 | 15277 |
| *hokC* | JW5002 | - | 16772 | 16900 |
| *nhaA* | JW0018 | + | 17492 | 18634 |
| *nhaR* | JW0019 | + | 18718 | 19599 |
| *rpsT* | JW0022 | - | 20836 | 21075 |
| *yaaY* | JW5003 | + | 21184 | 21378 |
| *ribF* | JW0023 | + | 21410 | 22327 |
