# Supplementary material for: A restriction-free method for gene reconstitution using two single-primer PCRs in parallel to generate compatible cohesive ends
Source: BMC Biotechnol. 2017 Mar 17;17:32. doi: 10.1186/s12896-017-0346-5 (PMC5356277; doi:10.1186/s12896-017-0346-5)
Supplement: Additional file 6: Table S3. — 15 kb DNA fragments from E.coli genome. (DOCX 13 kb) [file 12896_2017_346_MOESM6_ESM.docx]

**Table S3. 15 kb DNA fragments from *E.coli* genome**

| Gene | JW ID | Direction | Left nt | Right nt |
| --- | --- | --- | --- | --- |
| *surA* | JW0052 | - | 53437 | 54699 |
| *imp* | JW0053 | - | 54776 | 57106 |
| *djlA* | JW0054 | + | 57367 | 58158 |
| *yabP* | JW0055 | + | 58477 | 59103 |
| *yabQ* | JW0056 | + | 59124 | 59258 |
| *rluA* | JW0057 | - | 59708 | 60343 |
| *hepA* | JW0058 | - | 60379 | 63261 |
| *polB* | JW0059 | - | 63450 | 65777 |
| *araC* | JW0063 | + | 70390 | 71244 |
